# Identification of Fracture Mechanic Properties of Concrete and Analysis of Shear Capacity of Reinforced Concrete Beams without Transverse Reinforcement

**DOI:** 10.3390/ma13122788

**Published:** 2020-06-20

**Authors:** Oldrich Sucharda

**Affiliations:** Department of Building Materials and Diagnostics of Structures, Faculty of Civil Engineering, VSB-Technical University of Ostrava, Ludvíka Podéště 1875/17, 708 00 Ostrava-Poruba, Czech Republic; oldrich.sucharda@vsb.cz; Tel.: +420-597-321-382

**Keywords:** concrete, steel, reinforced concrete, beam, mechanical parameters, bending test, shear, inverse analysis

## Abstract

The study of new and innovative quasi-brittle materials offers new possibilities for use in construction, but detailed knowledge of their behavior and mechanical properties is required. The use of new materials in the design solution of a structure is usually associated with numerical methods, which has a number of both advantages and disadvantages. Sophisticated numerical methods, without a sufficiently detailed input knowledge, can provide highly variable results with little informative value. The main goal of this article is to present the procedure for the identification of fracture mechanical parameters for a specific concrete with the use of developed inverse analysis combining multi-criteria decision analysis, stochastic modelling and nonlinear analysis. Subsequently, the identified mechanical parameters of concrete are used for the parametric study of shear resistance of structural beams without shear reinforcement, as an alternative or generalized approach to the study of damage to concrete and concrete structures. This research includes an experimental program using 24 reinforced concrete beams and a detailed determination of basic and specific mechanical properties during laboratory tests. The process of inverse analysis is illustrated in detail for the solved task. The use of nonlinear analysis for detailed failure modelling is based on a 3D computational model and a fracture plastic material model for concrete. Finally, the results of the experimental program and numerical modelling are discussed, leading to a number of conclusions.

## 1. Introduction

Concrete is among the most important materials [1] used in the construction industry as a structural element. However, concrete has specific mechanical properties and flaws, depending on the type of stress or load. Concrete has many applications and there is a variety of compositions and production technologies. Among the concrete variants that have been created are self-compacting high-performance concrete [2], ultra-high-performance concrete [3,4] and fiber-reinforced concrete [5,6]. These materials can be classified as quasi-brittle and their mechanical properties and behavior can be very different to other variants of concrete; therefore, it is necessary to investigate them [7,8].

The fracture mechanics [9] are fit for a broader and general description of the damage and behavior of concrete and quasi-brittle materials. Wider utilization of fracture mechanics for concrete and composites is limited by the availability of comprehensive information in databases or recommendations. The fracture mechanical parameters of materials can be influenced by many factors. As fracture mechanics is a frequent subject of research, much information can be found in the literature [9,10,11,12]. However, many limitations exist in the available information, such that it is not useful for the analyzed composite types, as the focus of research to date has been on typical concrete. The databases have a rather limited scope [9,10,11,12] and use limited sample sizes or geometry ranges. Tests were often conducted on concretes of different batches and age categories, in different environments or using different test procedures on samples of different types and dimensions. Inconsistencies can therefore be observed when comparing fracture behavior among samples. For that reason, the identification of fracture parameters then requires the application of sophisticated methods and adequate verification of the identified properties [6].

Since the direct determination of some parameters, for example, fracture energy or tension soften function [13], is experimentally very demanding, there is an option to use sophisticated methods during inversion analysis and use results from laboratory testing in combination with numerical modelling to identify unknown parameters.

The design approach of inverse analysis includes the utilization of a series of methods, in particular the use of a multi-criteria analysis [14] for the decision-making process of material characteristics identification in combination with a nonlinear analysis and stochastic modelling. There are many approaches to inverse analysis such as decision trees, network models, neural networks, balance models, fuzzy logic or multi-criteria decision analysis (MCDA) [14]. Information on fracture mechanical properties can be used for a detailed study of a selected research problem. However, analytical methods in design codes often use only limited examples with respect to recommendations and, in some cases, use none at all. Typical cases may include the repair or strengthening of reinforced concrete (RC) structures by fiber reinforced polymer (FRP) [15] and carbon fiber reinforced polymer (CFRP) [16,17].

The use of new materials in designs is usually associated with numerical methods [18], which have a number of advantages and disadvantages. Summary results of the research and a number of recommendations on concrete and concrete structures for numerical modelling can be found in *Model Code 2010* [19]. On this basis, there are several possibilities for the use of material models including the disturbed stress field model for reinforced concrete [20], microplane model [21] or fracture-plastic material model [22,23] for nonlinear analysis [24,25].

The use of nonlinear analysis and material models is also connected with other numerical methods, namely, the finite elements method [18], the method of continuous strips, and the method of boundary elements.

The main design criteria include the check for bending moment (concrete-compression, steel-tension), shear failure [26,27] or punching [28,29]. In the case of reinforced concrete structures without shear reinforcement, a correct understanding of the failure mechanisms [30,31,32,33] and determination of the resulting load capacity is essential.

Typical structures include not only reinforced concrete beams without shear reinforcement, but other more complicated cases [28,33] such as continuous slabs, beams, shells of retaining walls or foundations [31]. The solved area is also the issue of punching, local loads or supports. Within the research topic of shear failure, there are many approaches in design codes or theories with empirical formulas for estimating the shear strength of reinforced concrete beams without transverse reinforcement.

The experimental program and numerical modelling will focus on reinforced concrete beams without shear reinforcement. This research includes an experimental program with 24 RC beams with specialized concrete, where existing analytical approaches are not sufficiently applicable. The experimental program also includes a detailed determination of basic and specific mechanical properties through laboratory testing. The process of inverse analysis is illustrated for the solved task. The use of nonlinear analysis for detailed failure modelling is based on a 3D computational model and a fracture plastic material model for concrete.

## 2. Inverse Analysis

Requirements for the generalized specification of the behavior of quasi-brittle materials, such as concrete, in fracture mechanics are expressed by basic material parameters including compressive strength, modulus of elasticity, tensile strength, and by specification of softening and the energy required to fracture. In particular, in the case of the latter property, this value has a significant impact on the total fidelity of subsequent nonlinear analyses of building structures.

Since the direct determination of the specific energy to fracture for crack propagation is experimentally very demanding, there is an option to use sophisticated methods within the inverse analysis and use results from laboratory testing in combination with numerical modelling to identify unknown parameters [6]. The presented approach of inverse analysis includes the utilization of a series of methods including multi-criteria analysis [14,34,35] for the decision-making process of material characteristics identification, in combination with a nonlinear analysis [24] and stochastic modelling [36].

**Figure 1:**
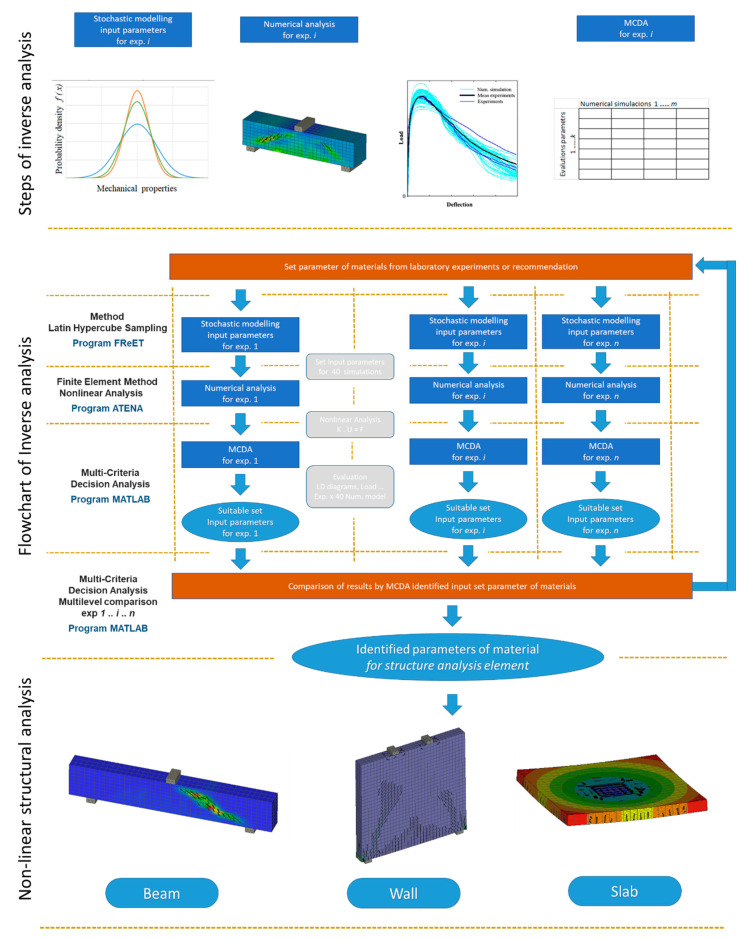
Flowchart of inverse analysis and structural analysis of structural elements.

The inverse analysis procedure is shown in Figure 1. Following the identification of the fracture mechanical parameters, a structural analysis for reinforced concrete beams, wall, slab and 3D reinforced concrete structures can be performed. The overall process of inverse analysis can be described in the following steps.

### 2.1. Stochastic Modelling

Key phases of the inversion analysis are stochastic modelling and the generation of input parameters for the identification of material characteristics. Specifically, the Latin hypercube sampling (LHS) [36] method and the FREeT (feasible reliability engineering tool) [37] program were used. Statistical characteristics were used for known mechanical properties from laboratory tests. For the identification of parameters at the beginning, estimated or known recommendations were used. The choice of the initial value affects the speed of convergence of the solution.

### 2.2. Finite Element Method and Nonlinear Analysis

Input data from stochastic modelling [36] were processed and a nonlinear analysis was produced with a suitable finite element model of the laboratory test, where the resulting developments of load-displacement diagrams were exported in summary. The numerical analysis was processed in the program ATENA (advanced tool for engineering nonlinear analysis) [38]. The generated input data and obtained load-displacement (LD) diagrams were processed using the multi-criteria analysis. The used deformation variant of the finite element method was based on the following solved equation:

(1) K⋅u=F

From the individual stiffness of finite elements K, vectors of unknown deformation of finite element u and the load vector of finite element F, the solution for the whole computing model was subsequently prepared. In the case of the nonlinear analysis and calculation using the method of finite elements, the resulting stiffness matrix of the structure is expressed as

(2) K=K(u).

For tensile softening in concrete, the form of Crack Opening Law [38] was used, in which the relationship is expressed as follows:

(3) $$\frac{\sigma }{f_{t}^{ef}}=\left\{1+{\left(c_{1}\frac{w}{w_{c}}\right)}^{3}\right\}\exp(-c_{2}\frac{w}{w_{c}})-\frac{w}{w_{c}}(1+c_{1}^{3})\exp(-c_{2})$$

This was subsequently based on the experiments modified as follows:

(4) $$w_{c}=5.14\frac{G_{f}}{f_{t}^{ef}}$$

where *w* is the crack opening, *w*_c_ is the crack opening at the complete release of stress, σ is the normal stress in the crack, *G*_f_ is the fracture energy and *f*_t_^ef^ is the effective tensile strength. 

### 2.3. Multi-Criteria Decision Analysis

The multi-criteria analysis [34,35] used was based on a criteria matrix consisting of individual criteria assessments for individual scenarios/calculations. The mathematical basis of the method can be expressed as

“*max*” *q* = *f*(*x*) = (*f*_1_(*x*),...,*f*_*k*_(*x*)) (5)

And

*q* ∈ *Q* = {*f*(*x*) : *x* ∈ *X*, *X* ⊆ *Rn*} (6)

where *X* is the possible set of variants and *x* are individual members of variables with the size vector *n*.

The calculation was based on the calculation of multipliers of *P_i_* criteria of each variant based on the information available. In addition, the multiplier of individual calculation variants was determined as

(7) $$U_{j}=\sum_{i=1}^{n}P_{i}\cdot w_{i}$$

The calculation must also include weights of individual criteria *w_i_*. The calculation continued with data normalization into dimensionless values. After setting up all members of the criteria matrix, the total multiplier of variants was calculated, from which the most suitable variant was selected as an identification result. A detailed description of the theory and application of the multi-criteria analysis is shown in [34,35].

The generated input data, obtained load-displacement (LD) diagrams of the numerical model and experiments were compared and processed using multi-criteria analysis in MATLAB. When there was no sufficient result (i.e., the result accuracy required was not met, criteria were not met), the whole process was repeated, assuming that up-to-date results were used and taken into account in the next input data generation. The evaluation of the identified values was carried out further on a multilevel comparison.

Other approaches to identify mechanical parameters included within inverse analysis are neural networks and stochastic modelling [39].

**Figure 2:**
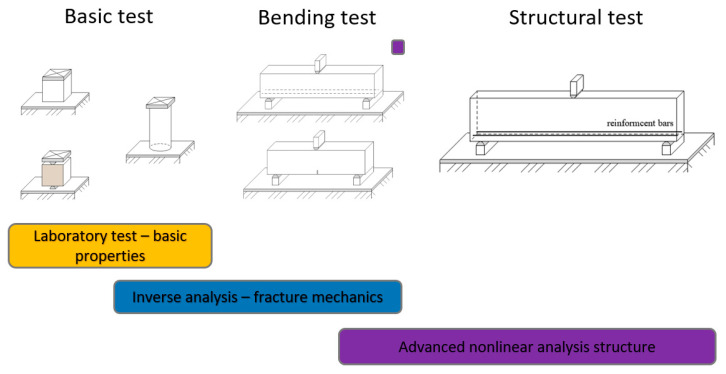
Experimental program: Basic test, bending test and structural test.

The basic concept of the experimental program and numerical modelling are shown in Figure 2.

## 3. Theoretical Aspect of Shear Failure Capacity and Analysis According to the Design Codes

**Figure 3:**
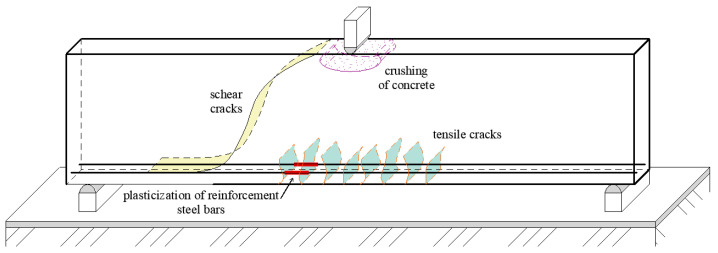
Typical failures of a reinforced concrete beam.

The specific problem of shear failure is selected for the numerical analysis of structural beams. For a closer understanding of the shear failure of reinforced concrete beams without transverse reinforcement, basic approaches based on design code or current research directions are presented. A typical failure of a reinforced concrete beam is shown in Figure 3.

**Figure 4:**
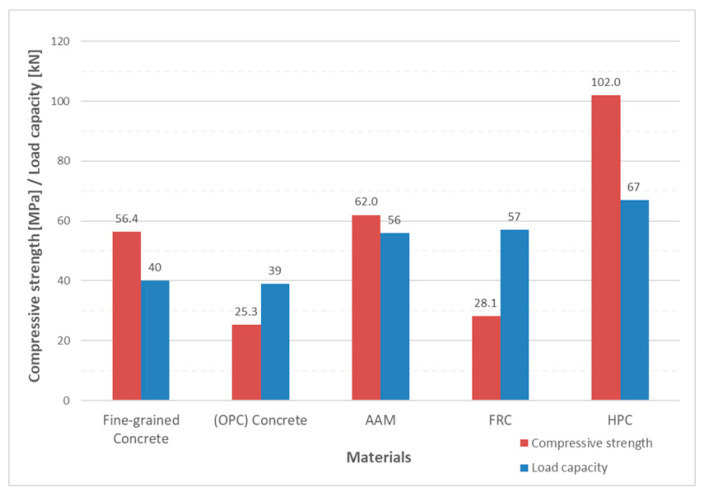
Comparison of load capacity of reinforced concrete beams and compressive strength.

The complexity of the problem of shear failure can be illustrated by comparing the total load capacity for the investigated type of beams in Figure 3, which is made of various concretes from the author’s previous research. All reinforced concrete beams from the experiments had shear failure. A detailed study of values in Table 1 and Figure 4 makes it possible to distinguish that a number of parameters that do not have the same effect enter the overall load capacity.

**Figure 5:**
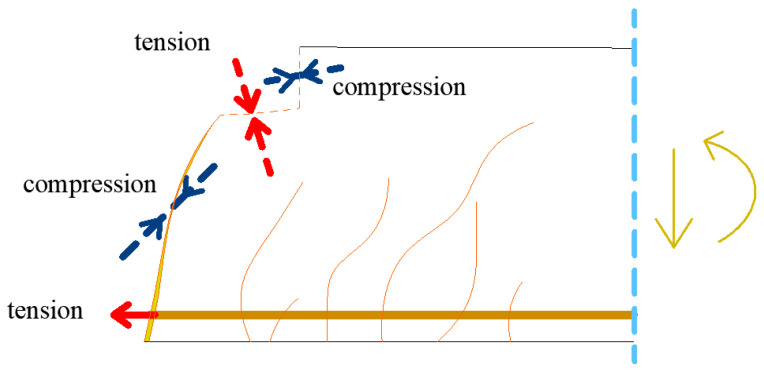
Potential shear transfer actions in beams without transverse reinforcement, adjusted according to [33].

Theoretical shear resistance and failure of beams can be formulated [33] by

(8) $$V_{R}=\int_{\xi =0}^{d´}\tau \cdot b\cdot d\xi =b\int_{\xi =0}^{d´}\tau \cdot d\xi ,$$

where *b* is the width of the cross-section, *d*’ is the depth of beam and *τ* is shear stress. The shear failure mechanism is shown in Figure 5 [33]. It can be seen from Figure 5 that the shear failure can be divided into part of a crack with tensile or compression areas. The actual formulation of the resulting shear load is further complicated by the steel reinforcement.

The current approach in *Eurocode 2* [43] sets the calculation of the shear load capacity without shear reinforcement according to (9), with a minimum value according to the relation (10).

(9) $$V_{R}=\left[C_{c}.k.{\left(100.\rho_{l}.f_{c}\right)}^{1/3}\right]b_{w}.d\leq V_{\min},$$

(10) $$V_{\min}=\left(0.035.\;k^{2/3}\sqrt{f_{c}}\right)b_{w}.d,$$

where *C*_c_ is the empirical coefficient of 0.18; *k* is the parameter that takes the size effect into account; *f*_c_ is the concrete´s compressive strength; *b*_w_ is the width of the cross-section; *d* is the effective depth of the cross-section; and $\rho_{l}$ is the reinforcement ratio for the longitudinal reinforcement. The minimum shear strength of reinforced concrete elements without transverse reinforcement solves the article [32].

New approaches in the solved area include the Critical Shear Crack Theory (CSCT) [33] from Professor Muttoni. The shear strength can be expressed as:

(11) $$V_{R}=b\cdot d\sqrt{f_{c}}\cdot f\left(w,d_{g}\right),$$

where *V*_R_ refers to the shear resistance, *b* to the width of the member, *d* to its depth, *f*c to concrete´s compressive strength, *d*_g_ to the aggregate size and *w* is the reference value of the critical shear crack opening. The ultimate shear force of beams and one-way slabs [31] without transverse reinforcement can be written as

(12) $$V_{R}=f_{ct}\cdot b\cdot d\cdot \left(v_{c}+v_{w}\right),$$

where *f*_ct_ is uniaxial concrete tensile strength, *b* is the width of the member, *d* is its depth, *v*_c_ is the parameter of compression chord and *v*_w_ is the parameter of cracked concrete web. The effect of cracked concrete web [31] can be written as

(13) $$v_{w}=167\frac{f_{ct}}{E_{c}}\left(1+\frac{2E_{c}G_{f}}{f_{ct}^{2}d}\right),$$

where *f*_ct_ is uniaxial concrete tensile strength, *d* is its depth, *E*_c_ is the modulus of elasticity of concrete and *G*_f_ is concrete fracture energy.

Among the known recommendations for the calculation of fracture energy for concrete is the *Model Code 2010* [19]:

(14) $$G_{f}=73f_{c}^{0.18},$$

where *f*_c_ is compressive strength. The formulation does not directly affect, for example, aggregate size or tensile strength. Other known relationships to VOS 1983 [38] include

(15) $$G_{f}=25f_{ct}$$

where *f*_ct_ is the uniaxial concrete tensile strength. From the new recommendation [44], we can note the relation from

(16) $$G_{f}=28f_{cm}^{0.18}\cdot d_{\max}^{0.32},$$

where *f*_cm_ is the mean value of the concrete compressive strength and *d*_max_ is the maximum aggregate size.

**Figure 6:**
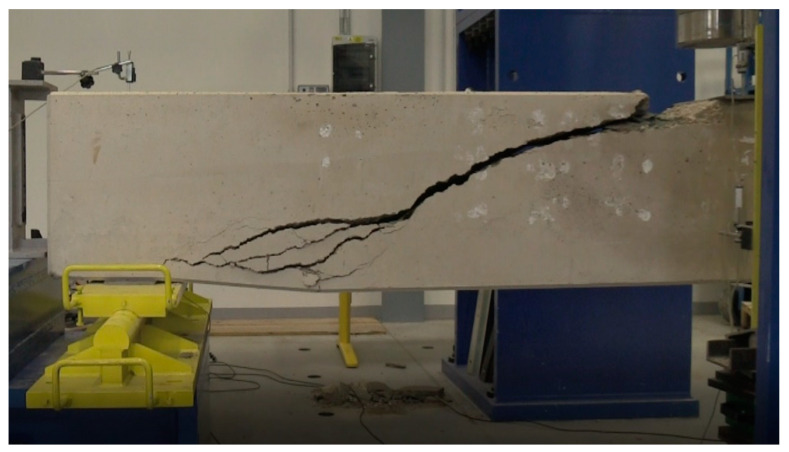
Reinforced concrete (RC) beams with transverse reinforcement (A1)—experimental program for alkaline-activated material (AAM).

**Figure 7:**
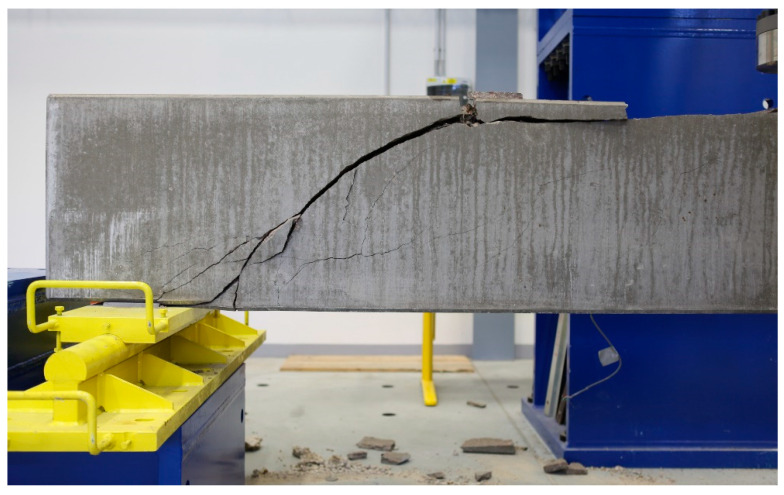
RC beams with transverse reinforcement (A1)—experimental program for material high-performance concrete (HPC).

The issue of shear failure is also relevant for reinforced concrete beams with shear reinforcement. Figure 6 and Figure 7 show the same type of beams (geometry, reinforcements), which differ only in the material used. These were type A1 beams according to the research program [45,46] and were 4.1 m long with a cross section of 300 × 550 mm. Figure 6 shows a beam with an alkaline-activated material (AAM) [41]. Figure 7 shows a reinforced concrete beam with high-performance concrete (HPC) [42]. In the case of the reinforced concrete beam with high-performance concrete (HPC) [42], the shear failure was located in one significant crack. The collapse of the reinforced concrete beam was sudden. A wider mesh of cracks formed in the reinforced concrete beam with alkaline-activated material (AAM) [41]. It was not until just before the collapse that the crack was significantly localized and gradually collapsed the beam.

In summary, the research of shear failure is often based on simplified test schemes that are not suitable for complex reinforcement concrete structures or specific concretes. These research approaches differ in the resulting constructive formulations, but also in the input parameters, the interpretation of the failure mechanisms and the control shear transmissions. There is no general consensus on solving a research task where the resulting load-bearing capacity is often very different.

There are a number of other theoretical approaches that vary by conciseness and amount of information required. However, it is clear from the above that it is appropriate to apply a generalized solution in the area of shear failure, which can be represented by advanced numerical modelling with 3D computational models [4,24]. Numerical modelling, however, must be based on the quasi-brittle nature of concrete [47] using nonlinear analysis and fracture mechanics.

## 4. Experimental Program

The experimental program included a comprehensive set of basic and specialized laboratory tests, followed by a comprehensive series of reinforced concrete beams without transverse reinforcement with differing degrees of reinforcement. The specific fine-grained, Baumit ProofBeton ©, with a fine aggregate up to 4 mm was found to have the following characteristics: It was waterproof, frost-resistant and with salt, and suitable for contact with drinking water [48]. The basic tests included compressive strength tests for cubes and cylinders, split tensile strength and modulus of elasticity. The basic tests were followed by bending tests of small beams. Two variants were selected: A typical variant included a three-point bending test with a notch; and a specific test of small reinforced concrete beams, which was intended for further evaluation within the inverse analysis. In addition to comparing the results with the laboratory program, recommendations for the calculation of the modulus of elasticity and tensile strength were selected. Great attention has been paid to research on the relation between [49] the modulus of elasticity and the compressive strength, as well as the split tensile strength of concrete [50,51,52,53,54,55,56,57,58,59,60]. The general relationship for split tensile strength and modulus of elasticity as a function of compressive strength are most frequently determined using Equations (17) and (18):

(17) $$f_{ct,st}=k.f_{c}^{n},$$

(18) $$E_{c}=\alpha .f_{c}^{\beta },$$

where *f*_c_ is the compressive strength, and *k*, *n*, α, and *β* are non-dimensional coefficients, which can be found in Table 2.

To use the relations in Table 2, it is important to note that the split tensile strength tests were carried out on cylinders in most cases. The relations stated were determined and based on an assessment of the extensive set of tests, where each relation also reflected the local conditions and specifics of the test programs. The coefficient *k*, within the interval 0.2–0.4, and the coefficient *n*, within the interval 0.6–0.8, are usually used for the split tensile strength. In the case of the modulus of elasticity, the difference in these coefficients is greater. The relationship of the modulus of elasticity as a function of the compressive strength is often limited and distorts the results. The quality of the aggregates and the water–cement ratio also play a role.

**Figure 8:**
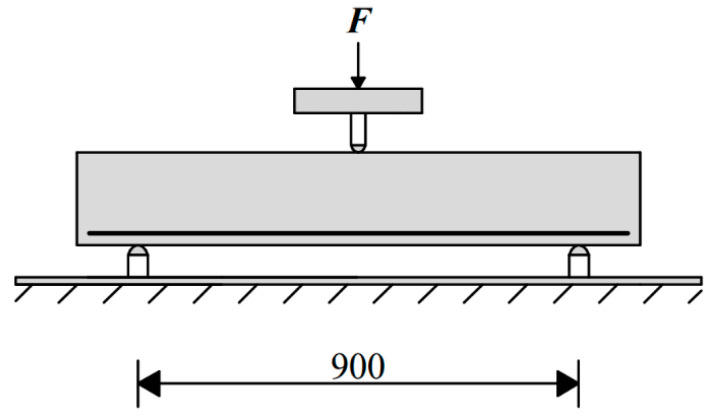
Reinforced concrete beam without transverse reinforcement—scheme of the test.

The main test program involved 24 reinforced concrete beams without transverse reinforcement. Four series are chosen for testing that differ in their degree of reinforcement, where each series had six beams. Specifically, the reinforced concrete beam was tested in a three-point bending test. The beam had a rectangular cross-section of 100 × 190 mm and a typical span of 900 mm. The diagram of the three-point bending test is shown in Figure 8. The beams were reinforced by two cross sections of the B500 concrete reinforcement at the bottom edge. The reinforcement cover was 20 mm. The reinforced concrete beam reinforcement for series one to four was 2 × ø6 B500, 2 × ø8 B500, 2 × ø10 B500 and 2 × ø12 B500, respectively. The objective of the laboratory tests was to evaluate the load displacement diagram during the loading test. Vertical deflection of the beams was measured in the middle of the span at the lower surface by means of extensometers. The beam was loaded with a deformation load.

For the sake of clarity, the overall summary of basic, bending and structural tests [61,62,63,64] is shown in Table 3.

## 5. Results for the Basic Test of Mechanical Properties

Summary results from the basic tests [61,62,63,64] are shown in Table 4. The range of laboratory tests for compressive and split strengths resulted in more detailed statistical characteristics being described. The compressive strength of the concrete for the cubes was 56.47 MPa; the statistical characteristics are shown in Table 5. The resulting standard deviation was 3.58 MPa for test 30 and the coefficient of variation was 0.06. The split tensile tests showed a greater dispersion of results: The average split tensile strength was 3.07 MPa with a standard deviation of 0.33 MPa and the coefficient of variation was 0.11. The average compressive strength for cylindrical was 48.85 MPa. The ratio between cylindrical and cubic strength was 0.865; this value is close to the recommended ratio of 0.85. The static modulus of elasticity, which had a value of 27.13 GPa, was measured for selected samples.

Fracture energy is an important mechanical parameter but its laboratory determination is difficult and there are more test methods that give different results. However, on the basis of laboratory tests, a comparison was made from the recommendations in Table 6. A reduction coefficient of 0.9 was used to convert the split tensile strength to the uniaxial strength. The resulting values range from 69 to 147 N/m. However, the relationships used have limited applicability here as they are for typical concretes.

## 6. Results for the Specialized Test of Mechanical Properties and Recommendation

Three control tests were performed to verify the aptness of the tensile strength calculation in the three-point bending test with a 25-mm notch; the results are shown in Table 4. The bending tensile strength was 3.14 MPa. The results from the compressive strength tests for the cylinders were to carry out a comparison with the recommendations given in Table 2. The results are summarized in Table 7.

**Figure 9:**
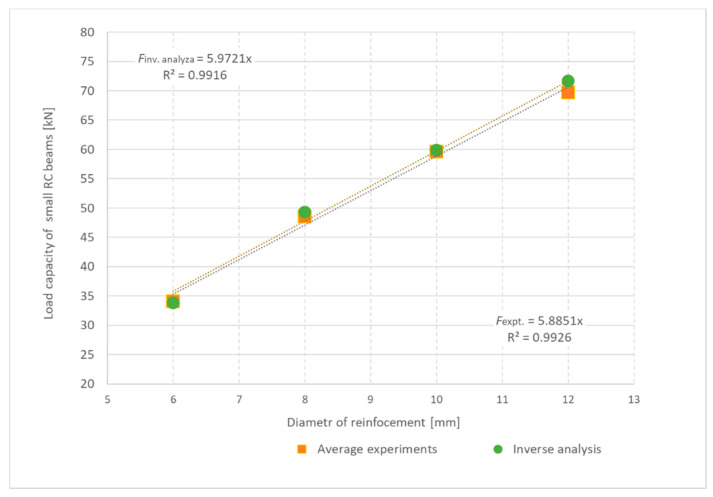
Load capacity of small reinforced concrete beams without transverse reinforcement: Experiments and inverse analysis results shown.

A total of twelve tests were performed in the three bending tests of small reinforced concrete beams with reinforcement, with three beams used for each test series. The resulting bearing capacity is evaluated graphically in Figure 9. The differences in load-bearing capacities in the individual series of reinforcement beams were usually small, although the differences were greater for the 10 mm diameter reinforcement. With a larger reinforcement diameter, the overall load-bearing capacity increases.

**Figure 10:**
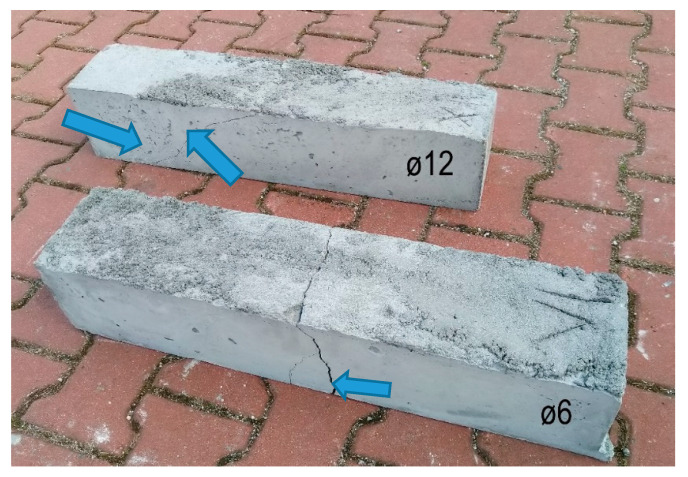
Small RC beams without transverse reinforcement—three-point bending test, with a span of 600 mm.

As an example, in Figure 10, a beam with a 6 mm diameter reinforcement and a beam with a 12 mm reinforcement are selected. For small beams with a 6 mm diameter reinforcement, it is apparent that the failure mechanism corresponds more to tensile failure. A beam with a diameter of 12 mm exhibited the failure mechanism of a typical shear crack.

**Figure 11:**
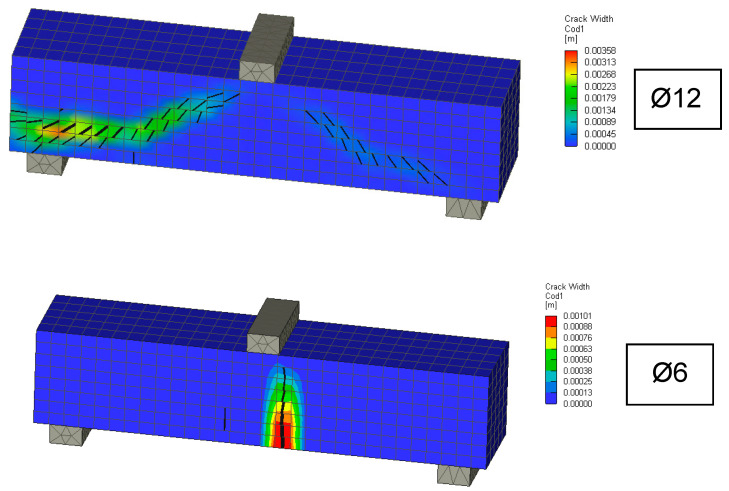
Small RC beams without transverse reinforcement, showing a 3D numerical model with a crack (min. 0.2 mm).

Results from the small reinforced concrete beams were used in the inverse analysis to determine fracture energy. For the fine-grained concrete mix used, the fracture energy relations (14–16) are unsuitable. The procedure for inverse analysis described in Section 2 was used. The identified parameter was fracture energy. The remaining mechanical properties of concrete were taken from laboratory tests. A reduction coefficient of 0.9 was used to convert the split tensile strength to the uniaxial strength. To verify the accuracy of the inverse analysis, computer simulations of 3D computational models with cracks are shown in Figure 11. During the modelling, the recommendations in [24] were respected and a fracture plastic material model for concrete [23] was used. The identified value of fracture energy is 44.3 N/m, and this was used for the calculation indicated by the green tick in Figure 9. The summary material characteristics for concrete and material model are given in Table 8.

A comparison of the experiment with numerical calculations adopting a different fracture energy for the selected small beam with the reinforcement diameter 12 is given in Table 9.

## 7. Parametric Analysis of Shear Capacity of Reinforced Concrete Beams

The main part of the research and experimental program was focused on the structural elements of reinforced concrete beams without transverse reinforcement. However, unlike typical analytical models in the recommendations and design codes, the shear crack theory analysis and stage was solved by advanced 3D numerical modelling that respects the propagation of cracks in concrete. The experimental program included four series of beams, where the reinforcement cross-section was from 0.3% to 1.2%.

**Figure 12:**
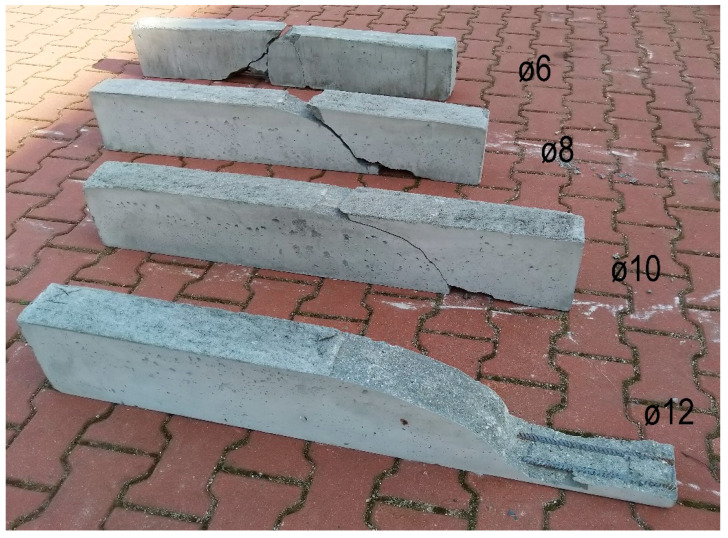
Reinforced concrete beams without transverse reinforcement after testing.

The reinforced concrete beams were reinforced with 6, 8, 10 and 12 mm diameter reinforcements. The specifics of the research program are that each series included 6 beams. The range was chosen because the tensile strength of the concrete is also important for the failure mode., i.e., a greater dispersion of the shear failure results can be expected. In general, the tensile strength has a wider range of values, as verified in Table 4. Consequently, this also allows for a statistical evaluation of the results by determining the standard deviation and coefficient of variation. The results are summarized in Table 10. The total load-bearing capacity of beams was from 26 kN to 49 kN. With higher reinforcement, the overall load-bearing capacity increased. However, this does not apply to all experiments. For example, the maximum value (41 kN) was for a reinforcement diameter of 8 mm and the minimum value (38 kN) was obtained with a reinforcement diameter of 10 mm. An overview of the four types of beams after the test is shown in Figure 12. A shear failure can be clearly seen in the beams with 8 to 12 mm diameter reinforcements. For a beam with diameter 6 mm of reinforcement, the failure mode was different. In this beam, a vertical tensile crack in the middle of the lower edge of the beam and a shear crack are visible.

**Figure 13:**
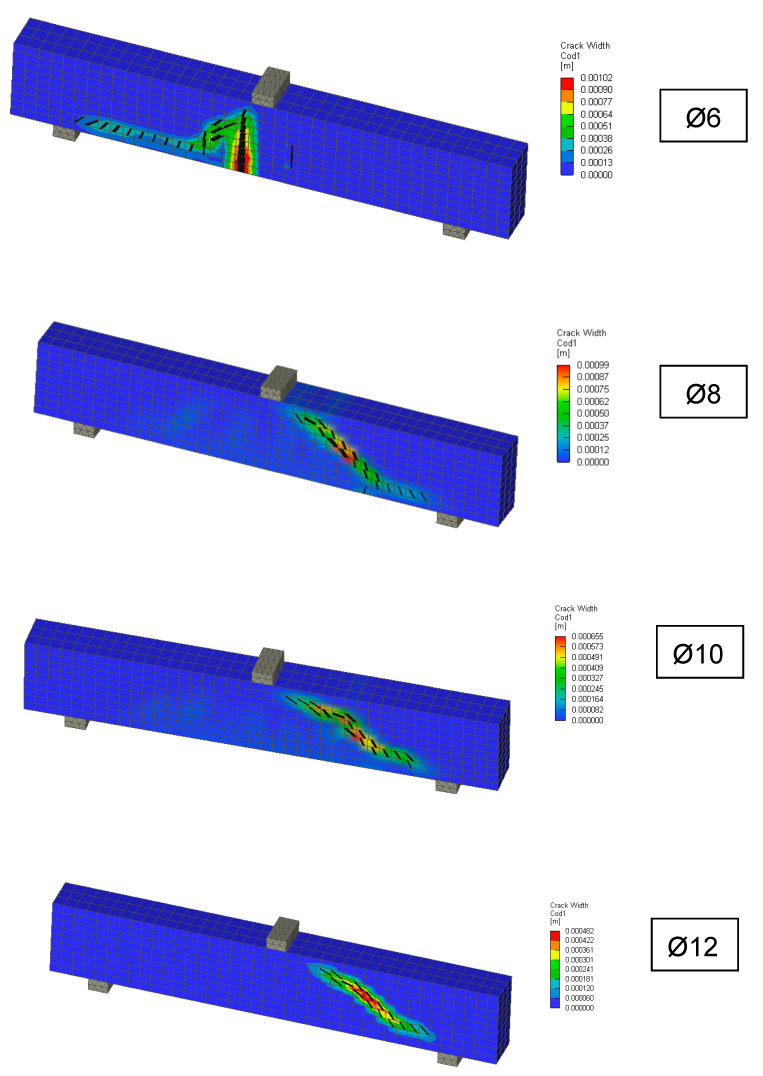
Reinforced concrete beams without transverse reinforcement—3D models with crack (min. 0.2 mm).

**Figure 14:**
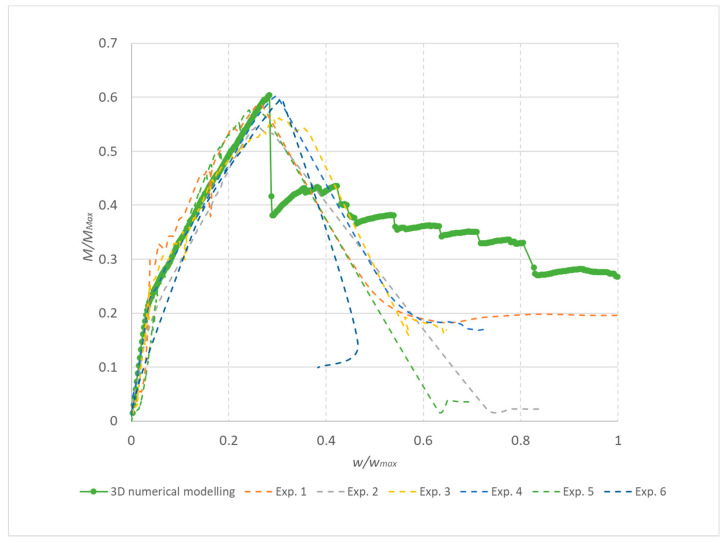
Results for reinforced concrete beams without transverse reinforcement, reinforcement 10 mm: Experiment 1–6 and 3D model.

The range of coefficients of variation was between 3% and 6% for the individually reinforced type concrete beams and the average value was 5%. Following this, 3D numerical modelling of experiments was carried out using the identified mechanical properties of concrete. Numerical simulations, calculation parameters, and the calculation model differed only by the reinforcement used. The individual computational models are shown in Figure 13. It is evident that the failure mode was very similar for numerical models and experiments. In addition, the numerical model makes it possible to obtain the results of a maximum crack: The crack width was from 0.48 mm to 1.02 mm. Figure 14 shows the results of the load capacity diagram for experiments and 3D numerical simulation for reinforced concrete beams with a reinforcement diameter of 10 mm. The results are related to the maximum theoretical bending moment *M*_max_ in the cross section and the relative deformation calculated to the maximum deformation of *w*_max_ 1/200 of the span of the beams. Figure 14 shows the initiation of linear loading. Subsequently, a shear crack develops in the reinforced concrete beams and gradually propagates. In the area of shear crack propagation, the results show greater dispersion of stiffness. The test is terminated by a significant crack opening and a decrease in force. The loading of the beams proceeded by controlled deformation.

**Figure 15:**
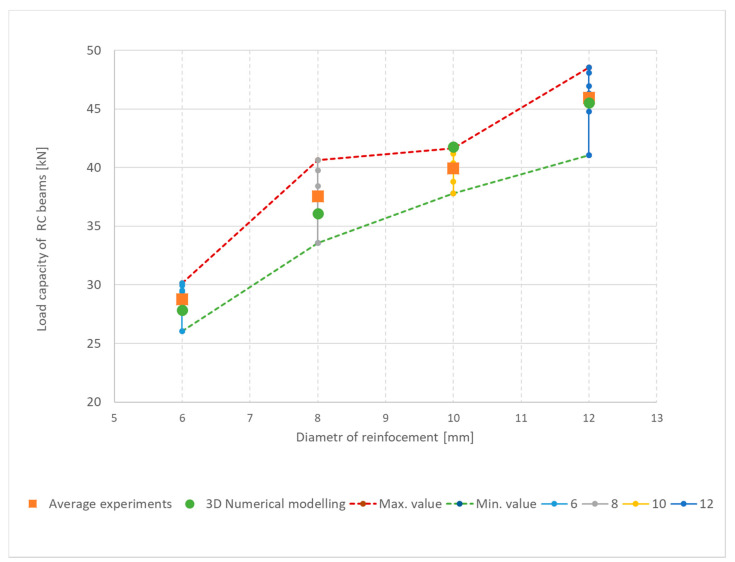
Summarized results for load capacity of reinforced concrete beams without transverse reinforcement.

Larger cracks were found in beams with smaller reinforcement diameters. A summary of the results is provided in Table 10. The greatest difference between the calculation and the experiment was 4.1% and the average difference was 1%. The results of experiments and numerical simulations are also plotted in Figure 15.

## 8. Discussion

Based on the experimental program, it was found that the average compressive strength of 48.85 MPa on cylinders was in the usual ratio to the compressive strength of 56.47 MPa on cubes: The obtained ratio of 0.865 is very close to the usual ratio of 0.85. The influence of fine-grained concrete aggregates had no significant influence on the shape of the test body used. The determined split tensile strength and its recalculated uniaxial concrete tensile strength were less than most recommendations. A reduction coefficient of 0.9 was used for the conversion of strengths. Considering the cube compressive strengths and the split tensile strength, it can be concluded that the coefficient of variation was significantly higher for the split tensile strength. In both cases, however, the test set values were in the interval quantile 0.01–0.99. The fracture energy parameter is also related to uniaxial tensile strength. Comparing the recommendations for the calculation of fracture energy and the results of the inverse analysis, it is clear that the use of formulas for typical concrete is usually not appropriate: The difference in the resulting beam load capacity and the experiment can be up to about 47%. The use of inverse analysis to identify the fracture energy parameter has proved to be a more suitable approach.

The analysis process itself should take into account and meet the following criteria:

- Ensure there is a sufficient set of basic mechanical properties (compressive strength, transverse tensile strength, modulus of elasticity).
- Appropriately select specialized tests for direct or indirect determination of specific parameters (e.g., fracture energy) or use recommendations. Small reinforced concrete beams were used to solve this specific problem. The sensitivity of the fracture mechanical parameters themselves is broadly discussed in [40].
- If inverse analysis is used, select appropriate methods so that results are sufficiently reliable. Among the possible methods belongs the presented method of combining multi-criteria decision analysis (MCDA) [14], stochastic modelling [36,37] and nonlinear analysis [22].
- The use of numerical modeling and nonlinear analysis requires the appropriate selection of the material model and adherence to the recommendations for nonlinear solutions. Calculation parameters include type of finite element, finite element mesh, a mathematical solver, and conversion criteria. The modelling in this study was based on the recommendations in [24].

The resulting fracture energy identified was 44.3 N/m. Numerical modelling and shear failure analysis were performed based on a comprehensive set of mechanical properties of the concrete used. 3D numerical modelling made it possible to respect the propagation of cracks in concrete.

**Figure 16:**
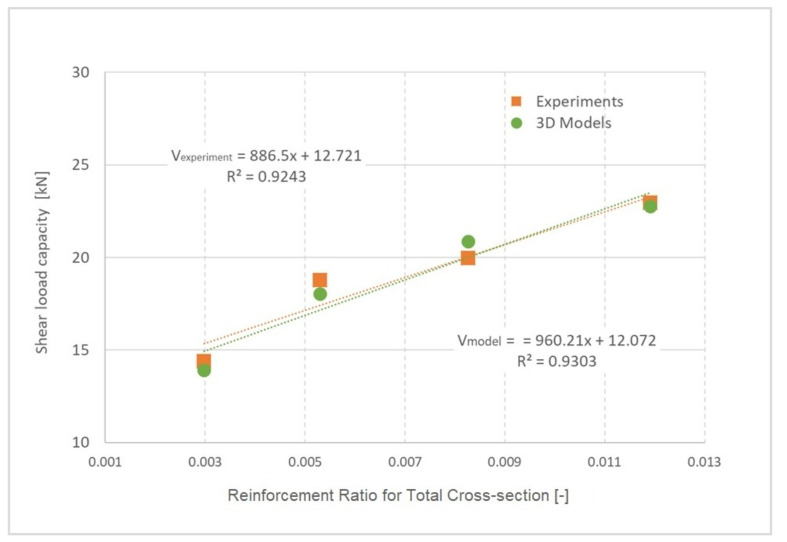
Shear load capacity of reinforced concrete beam without transverse reinforcement.

The results of tests and numerical modelling can be interpreted in the form of shear capacity of the beam in Figure 16, depending on the degree of cross-section reinforcement. A shear load regression function was also determined for experiments and numerical models, as shown in Equations (19) and (20):

(19) $$V_{experiment}=886.5x+12.721,\;\left(R^{2}=0.9243\right)$$

(20) $$V_{model}=960.21x+12.072,\;\left(R^{2}=0.9303\right)$$

where *x* is the degree of cross-section reinforcement. In both cases, the confidence level is greater than 92%. The predicted shear capacities and failure of numerical 3D models can be considered very good.

## 9. Conclusions

This research presents an approach to the determination of fracture mechanical parameters for specialized fine-grained concrete for which it is not possible to use the available recommendations. The presented inverse method combines multi-criteria decision analysis (MCDA), stochastic modelling with Latin hypercube sampling (LHS) and nonlinear analysis.

Part of the research and experimental program was focused on the determination of load capacity of reinforced concrete beams without transverse reinforcement and modelling of shear failure. However, knowledge of detailed mechanical parameters of concrete is necessary to gain an understanding of the problem; especially in the case of specific and high-performance concretes, the mechanism of damage and behavior may differ compared with typical concretes. This case was solved by this research, which was a specialized fine-grained concrete with aggregates up to 4 mm. Numerical modelling with a 3D computational model and fracture-plastic material model was used for determination of shear resistance of the beam. This approach made it possible to effectively describe the overall load-bearing capacity as well as the mechanism of failure and collapse of the beam itself. The following conclusions can be drawn:

- Laboratory tests of compressive strength and split tensile strength for the specialized concrete show that the coefficient of variation is nearly two-times greater for the transverse tensile strength.
- The split tensile strength for the fine-grained concrete mixture was significantly lower than foreseen in the recommendations.
- A well-known coefficient of 0.85 works well to convert between compressive strengths for cubes and cylinders.
- The modulus of elasticity is significantly smaller than indicated in most recommendations and research.
- In the case of fracture energy determination, inverse analysis can be used to identify it, but this requires the use of a specialized test program and numerical methods. Recommendations for the calculation of fracture energy can by only usually intended for typical concretes;
- The chosen approach to shear failure modelling allows for the load capacity and failure mechanism to be correctly understood. Complex mechanical properties of concrete and numerical modelling can then be used for more complex structures that cannot be represented by analytical models.

Another benefit of the approach presented for the analysis and modelling of concrete structures is that it enables the task to be further extended by the problems of durability and reliability of structures. In further research, the author will focus on the optimization of the method for the identification of mechanic parameters and the possibility of using fiber-reinforced concrete for beams without shear reinforcement, as this research is related to the solved problem.

## Figures and Tables

**Table 1:** Load capacity of reinforced concrete beams.

Material	Fine-Grained Concrete	Concrete	Alkaline-Activated Material (AAM)	Fiber-Reinforced Concrete (FRC)	High-Performance Concrete (HPC)
*f*_c,cube_ [MPa]	56.4	25.3	62.0	28.1	102.0
*f*_t, split tension test_ [MPa]	3.0	2.2	3.4	3.6	5.8
*E*_c_ [MPa]	27.1	18.5	26.3	18.3	42.1
Maximum aggregates [mm]	4	16	16	16	16
Reinforcement Ratio for Total Cross-section [-]	0.0083	0.0083	0.0083	0.0083	0.0124
Reference	-	[40]	[41]	-	[42]
Load [kN]	40	37–41	56	57	67

**Table 2:** Coefficients *k, n,* *α, β* [49].

Source	*k*	*n*	*α*	*β*
JCI [50]	0.13	0.85	6.3	0.45
JSCE [51]	0.44	0.5	4.7	0.5
JSCE [52]	0.23	0.66	9	0.33
AIJ [53]	0.18	0.75	8.56	0.33
CEB-FIB [54]	0.3	0.66	9.5	0.33
ACI318-11 [55]	0.56	0.5	4.73	0.5
Raphael [56]	0.313	0.66	-	-
Gardner [57]	0.33	0.66	3.24	0.63
Oluokun et al. [58]	0.216	0.79	-	-
Oluokun [59]	0.2	0.7	-	-
Arioglu et al. [60]	0.387	0.63	-	-

**Table 3:** Test setup program.

Test	Sample	Dimensions of Body [mm]
Compressive test—cube	30	150 × 150 × 150
Compressive test—cylinder	6	150 × 300
Modulus of elasticity	3	150 × 300
Split tension test	30	150 × 150 × 150
Three-point bending test	3	150 × 150 × 600 (span 500, notch 25)
Bending test of small reinforced concrete beams	12	150 × 150 × 700 (span 600)
Reinforced concrete beams without transverse reinforcement	24	100 × 190 × 1150 (span 900)

**Table 4:** Material properties of basic tests.

Material Properties	Mean [MPa]	Standard Deviation [MPa]
Compressive strength-cube	56.47	3.58
Compressive strength-cylinders	48.85	1.08
Split tension strength	3.07	0.33
Bending tension strength	3.14	0.29
Modulus of elasticity	27,130	960

**Table 5:** Material properties of compressive strength and split tension tests.

Material Properties	Compressive Strength [MPa]	Split Tension Strength [MPa]
Average	56.47	3.07
Minimum	50.31	2.42
Maximum	64.63	3.70
Quantile 0.01	48.16	2.31
Quantile 0.025	49.45	2.43
Quantile 0.05	50.56	2.53
Quantile 0.95	62.38	3.61
Quantile 0.975	63.49	3.71
Quantile 0.99	64.78	3.83
Standard deviation	3.58	0.33
CoV [–]	0.06	0.11

**Table 6:** Material properties of fracture energy [N/m].

VOS 1983 [38] (14)	Model Code 2010 [19] (15)	Mari et al. [44] (16)
69	147	88

**Table 7:** Results of mechanical properties *f*_ct,st_ and *E*_c_ for coefficients *k, n,* *α,β.*

Source	*f*_ct,st_ [MPa]	*E*_c_ [GPa]
JCI [50]	3.54	36.25
JSCE [51]	3.08	32.85
JSCE [52]	2.99	32.48
AIJ [53]	3.33	30.89
CEB-FIB [54]	3.91	34.28
ACI318-11 [55]	3.91	33.06
Raphael [56]	4.08	-
Gardner [57]	4.30	37.54
Oluokun et al. [58]	4.66	-
Oluokun [59]	3.04	-
Arioglu et al. [60]	4.48	-

**Table 8:** Results of mechanical properties for concrete.

Material Properties	Value	Units
Compressive strength—cube	56.47	MPa
Compressive strength—cylinders	48.85	MPa
Modulus of elasticity	27.13	GPa
Tensile strength	2.76	MPa
Fracture energy	44.3	N/m
Maximum aggregate	4	mm

**Table 9:** Load capacity [kN] of small reinforced concrete beam without transverse reinforcement (diameter 12 mm).

VOS 1983 [10] (12)	Model Code 2010 [28] (13)	Mari et al. [36] (14)	Inverse Analysis	Experiments (Average)
82	103	89	72	70

**Table 10:** Results of reinforced concrete beam without transverse reinforcement: Experiments—load capacity [kN].

**Reinforced of Diameter [mm]**	2 × 6	2 × 8	2 × 10	2 × 12
**Reinforcement Ratio for Total Cross-section [–]**	0.003	0.012	0.008	0.012
**Sample [Sample]**	6	6	6	6
**Maximum [kN]**	30	41	42	49
**Minimum [kN]**	26	34	38	41
**Standard Deviation [kN]**	1.47	2.35	1.32	2.51
**VoC [-]**	0.051	0.062	0.033	0.055
**Average of Experiments [kN]**	29	38	40	46
**3D Model** **—** **Simulations**	28	36	42	46
**Experiment/3D Model**	1.033	1.041	0.956	1.009
